# Optic Pit Maculopathy

**Published:** 2017

**Authors:** Ovidiu Musat, Stella Ioana Popescu, Corina Cristina Cernat, Ana Maria Boariu, Lucian Alexandru

**Affiliations:** * Ophthalmology Department, “Dr. Carol Davila” Central University Military Emergency Hospital, Bucharest, Romania

**Keywords:** optic pit, vitrectomy

## Abstract

This paper discusses a 32-year-old woman’s case who presented a chronic decrease of visual acuity.

In this case, the treatment was complex and consisted of a 25 GA vitrectomy, MLI peeling, covering the defect at the optic nerve papilla with that patch, gas endotamponade (C3F8).

**Abbreviations:** MLI = internal limiting membrane; ODP-M = optic disc pit maculopathy

## Introduction

Optic nerve transmits visual information from the retina to the lateral geniculate body. The optic nerve consists of retinal axons that are second-degree neurons of the afferent visual pathway. This makes the optic nerve part of the central nervous system not a peripheral sensory nerve.

Optic nerve consists of about 1,2 million axons from the eye, 90% of the retinal ganglion cells being concentrated in the macula. About 80% of the optic nerve fiber originates from the macula, hence macula diseases can mimic optic nerve diseases and vice versa. 

Optic disc is a congenital defect that consists of the presence of a “crater like” depression in the optic nerve head.

Optic disc pits are thought to occur in approximately 1 per 10000 patients without sexual or racial predilection [**1**].

Most of the time, pits are found in the temporal level but can as well appear at any level of the disc [**2**]. They may be associated with adjacent peripapillary retinal pigment epithelial changes.

Pits present unilaterally and rarely more than one pit can be seen on a single optic disc. They can occur at any age but especially in young adults.

The lesion appears oval or round in shape and may be whitish or yellowish in color at the base.

The pathogenesis is not established [**3**].

Four possible sources have been described for intraretinal or subretinal fluid over the years:

1-the vitreous.

2-CSF (cerebrospinal fluid) which was suspected to enter the intra and sub retinal spaces from the subarachnoid space through the ODP defect.

3-leakage from blood vessels at the ODP was based on the finding of late hyperfluorescence at the ODP in fluorescein angiography as well as in the area of macular elevation in eyes with ODP-M.

4-fluid from the choroid, through the Bruch’s membrane and peripapillary atrophy.

## Case Report

We described a case of ODP-M in a young woman patient with a history of progressive reduction of central vision of the left eye of more than 1-month duration. No history of trauma was mentioned. 

Ophthalmic examination showed a best corrected visual acuity (BCVA) of 20/ 20 in her right eye and hand movements in her left eye. Intraocular pressure was normal (IOP 15 left eye; IOP 15 right eye). Dilated fundus examination of the left eye showed the characteristic aspect of ODP-M described above.

**Fig. 1:**
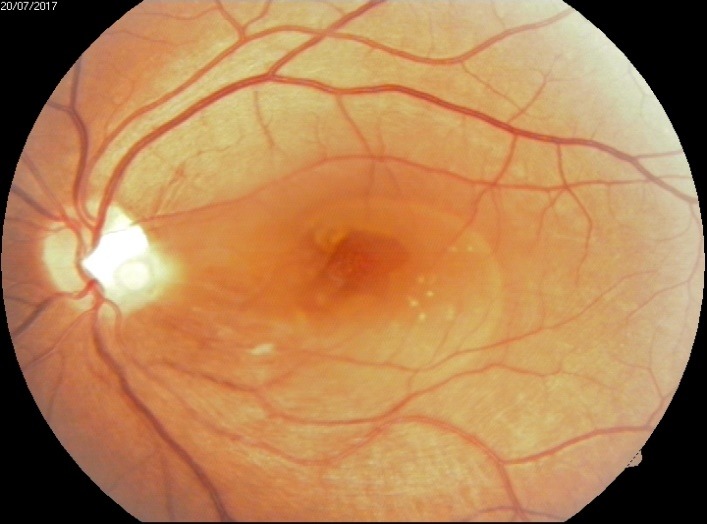
Optic Pit Maculopathy

Optical coherence tomography (OCT) with retinal map analysis of the left eye showed serous macular detachment.

**Fig. 2:**
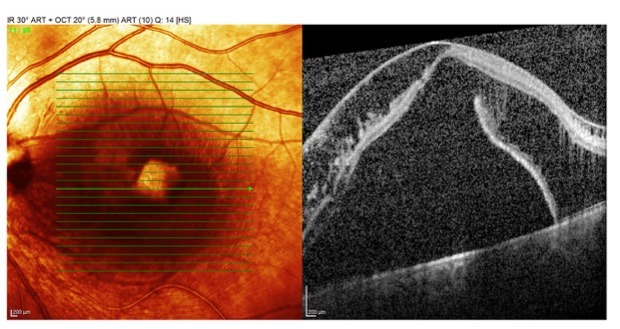
Preoperative OCT

The patient went to standardized pars plana vitrectomy (25G), peeling of the internal limiting membrane, defect covering with a patch of MLI and gas tamponade with favorable results.

**Fig. 3:**
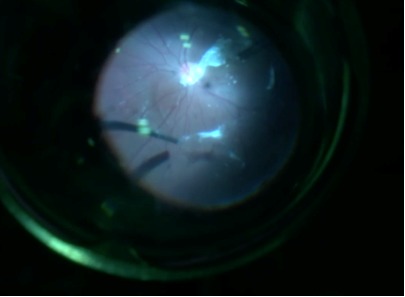
Peeling of hyaloid-intraoperative image

**Fig. 4:**
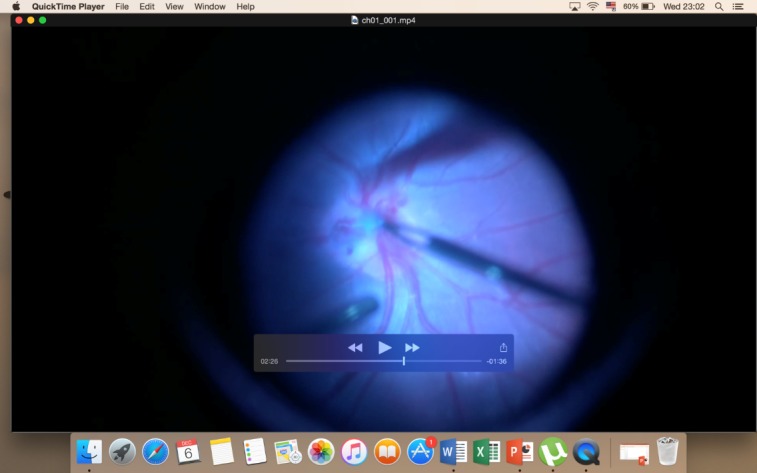
Extract of intraoptic pit membrane-intraoperative image

Two months after the surgery, the patient’s BCVA was 0,06 and retinal examination showed a reduction in the subretinal fluid documented both clinically and by OCT.

**Fig. 5:**
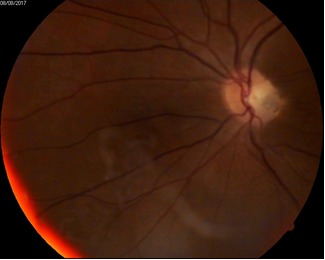
Postoperatory image - after 45 days

**Fig. 6:**
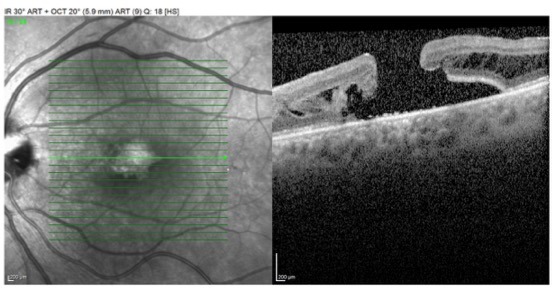
OCT image - after 45 days

## Discussion

In this case, the peeling of the internal limiting membrane was done so that to relax existing trains at that level and, finally yet importantly, to complete the papillary defect [**4**].

Peeling of MLI has been limited precisely in the idea of preserving the biological material for macular hole surgical cure. Laser has not been used in the interpapilomacular space in this first stage due to huge macular edema and of course to avoid functional alterations [**5**].

## Conclusion

Until today, there has been no guide to treating such challenging cases, difficult to manage. Current evidence suggests that the subretinal fluid associated with the maculopathy is most likely vitreous in origin.
